# Active for Life: A Work-based Physical Activity Program

**Published:** 2007-06-15

**Authors:** Beverly B Green, Allen Cheadle, Adam S Pellegrini, Jeffrey R Harris

**Affiliations:** Group Health Cooperative; University of Washington, Seattle, Wash; American Cancer Society, University Place, Wash; University of Washington School of Public Health and Community Medicine, Seattle, Wash

## Abstract

**Background:**

The American Cancer Society's Active for Life is a worksite wellness program that encourages employees to be physically active. This paper reports the experience of implementing Active for Life in a worksite setting and its longer-term impact on physical activity.

**Context:**

The Active for Life intervention was provided to employees at Group Health Cooperative, a nonprofit health care system in the Pacific Northwest with 9800 employees.

**Methods:**

Posters, newsletters, health fairs, and site captains promoted enrollment in Active for Life. Interventions included goal-setting, self-monitoring, incentives, and team competition. Preprogram and postprogram changes in physical activity were assessed at baseline, 10 weeks, and 6 months.

**Consequences:**

Active for Life was offered to 3624 employees, and 1167 (32%) enrolled; 565 (48%) completed all three surveys. At 10 weeks, all physical activity measures increased significantly. The proportion of employees meeting the guideline of the Centers for Disease and Control and Prevention for physical activity increased from 34% to 48% (*P* < .01). At the 6-month follow-up, the frequency of exercising enough to work up a sweat (*P* < .01) remained significantly increased, but other measures of physical activity declined toward baseline.

**Interpretation:**

A 10-week worksite program implemented at multiple facilities increased physical activity by the end of the intervention, but these changes were not sustained over time. Future interventions might include extending the length of the program, repeating the program, or adding larger economic incentives over time. Any such alternative models should be carefully evaluated, using a randomized design if possible.

## Background

Unhealthy lifestyle, including lack of physical activity, poor nutrition, and being overweight, is the second leading cause of preventable death after tobacco use (1). Yet more than 60% of American adults are not regularly physically active, and 25% are not active at all (2). Identifying strategies to increase physical activity and improve nutrition remains a major public health challenge. Because most adults spend half of their waking hours on the job, the workplace offers a promising setting for environmental, policy, and programmatic interventions to help people adopt more healthy lifestyles.

The American Cancer Society's Active for Life (AFL) is a 10-week, worksite-based program employing strategies that have been identified as effective by *The Community Guide* (3,4). These strategies include the use of incentives, a team approach, and the targeting of social norms related to physical activity. AFL was originally developed at the Centers for Disease Control and Prevention (CDC) as the Director's Challenge. An evaluation showed that 64% of CDC employees enrolled and 79% of the participants reached their activity goals. However, only 32% responded to both preprogram and postprogram surveys, and no longer-term follow-up was conducted (5). AFL has been licensed by the American Cancer Society (ACS) and implemented in a number of worksites, but replication of CDC's initial results and longer-term evaluation has not been performed.

This paper reports the experience of Group Health Cooperative (Group Health) in implementing AFL in 10 of its facilities (clinic, hospitals, and administrative units) and measures the longer-term impact on physical activity.

## Context

The setting for this intervention was Group Health, a nonprofit health care system in the Pacific Northwest. Group Health provides both medical coverage and medical care to 530,000 members. Seventy percent of the members receive care via a staff-model health care organization (i.e., physicians are employees of the HMO) at Group Health-owned facilities and from Group Health-employed staff.

Group Health, with 9800 employees in its staff-model health care organization, has considerable interest in improving the health of its employees for several reasons: 1) Group Health provides health insurance to its own employees; 2) healthy employees (including those more physically active) have less absenteeism and are more productive at work (6); 3) Group Health sought to improve employee morale at a time when implementing electronic medical records was associated with increased stress; and 4) it was hoped that adopting AFL for employees would provide a model for Group Health patients and the general public.

## Methods

### Program planning and implementation

The AFL program at Group Health took approximately 3 months to plan and implement (see Table 1 for a timeline). After support was secured from Group Health's Executive Officer Group, a steering committee was formed with employees from clinical quality and education, who provided leadership, implementation, Web page development and maintenance, operational coordination, and programmatic support; employees from communications, who were consulted about promotional materials and a plan for disseminating messages; and employees from the Department of Prevention, who collaborated with ACS to develop and implement an evaluation plan. AFL was offered at 10 Group Health facilities: six of 20 clinics (with 60 to 135 employees per clinic), both of the Group Health-owned hospitals (with 950 and 1300 employees), and two of three administrative facilities (with 780 employees and 60 employees). Group Health wanted to implement and evaluate the program before offering it throughout the organization. The participating facilities were selected because of expressed interest or an absence of competing priorities.

ACS provided AFL protocols and associated materials, assisted the project manager, and provided a 2-hour training session for the site captains (who were selected by the steering committee). The site captains encouraged enrollment, helped form teams and choose team captains, and assisted team captains in team management, point collection, and problem solving. Newsletters, e-mail messages, posters, word of mouth, and health fairs were used to notify employees of the start date and to encourage enrollment. Each team consisted of a team captain and four to eight participants from the same facility. An internal Web site developed for the program offered automated enrollment and tracking forms, resources such as motivational tips and links to other healthy lifestyle resources, and ongoing success stories from participants.

Each AFL participant set weekly goals for minutes of physical activity, earning one point for each minute. Participants were scored on goal attainment (recorded as a percentage of their goal) rather than absolute minutes of exercise. For example, a participant might have set a goal of walking for 40 minutes 5 days a week (200 minutes total per week) but only walked 150 minutes, yielding a score of 75%, or 75 points. If participants met their weekly goal, they were encouraged to set a higher goal for the following week. Employees also received extra credit points for eating at least 5 servings of fruits and vegetables a day, up to 25 points per week (for a maximum of 125 points weekly).

All participants received a pedometer with the Group Health logo. Participants who completed the program received athletic socks and bicycle lights. Other incentives included a team prize for the group that scored the highest points (a lunch and rope-jumping entertainment), individual awards for success stories, and eligibility for a drawing of several prizes (gift cards and one grand prize of a spa day) for those who completed evaluations. Progress of each team was tracked on the Group Health AFL Web site.

### Evaluation

Physical activity and other covariates were ascertained using a Web-based survey tool (SurveyMonkey) that issued up to two reminders for participants who failed to complete the survey. Self-reported physical activity was evaluated by three methods: exercise metabolic equivalents (METS) per week, frequency of sweating with exercise, and a stage of change question (Table 2). The Godin Weekly Leisure-Time Exercise Questionnaire measures weekly frequency of strenuous, moderate, and mild leisure-time activity of at least 15 minutes and produces a single score (exercise METS per week) (7). The Godin questionnaire has an additional question about sweating during exercise. The sweat question has a correlation coefficient of 0.56 compared to maximal oxygen uptake (VO_2_ Max) (8). The stage of change places activity level in five categories matching the stage of change for physical activity (see Table 2) (9). Other covariates included consumption of fruits and vegetables (measured using a single question developed for the Seattle 5 A Day worksite intervention) (10), satisfaction with work (using the question "All things considered, how satisfied are you with Group Health Cooperative as a place to work?" from Group Health's annual employee survey), and an additional question as to whether Group Health employees were encouraged to have a healthy lifestyle. Survey respondents were asked a series of questions at the 10-week follow-up for ranking perceived benefits, motivational factors, and barriers to participation, and one open-ended question that permitted comments to be added.

The analysis used *t* tests and chi-square tests to examine changes over time in the key outcome measures among AFL participants. To adjust for potential bias, we compared baseline characteristics of participants who completed all three surveys to those of participants who completed one or two. Logistic regression analysis was used to control for demographic and other variables that were associated with survey nonresponse. All analyses were performed using SAS (SAS Institute Inc., Version 8.0, Cary, NC).

Group Health and ACS leadership received a detailed report of the implementation processes and evaluation results of this pilot. Results were presented at several community and scientific meetings. Because this pilot was originally conducted as a program evaluation, it was not originally submitted to the Human Subjects Review Committee of Group Health. When we decided to seek publication of our experience and results, we did seek and receive approval from the committee.

## Consequences

AFL was offered to 3624 employees, and 1167 (32%) enrolled. Enrollment varied by facility type and averaged considerably higher in the six clinics (66.5%) than the two hospitals (20.4%) and the two administrative centers (40.2%). Of the enrolled participants, 811 (69%) reported at least 1 week of points to their team captain; 595 (51%) reported all 10 weeks of points. If captains were unable to complete their tasks, the team members were more likely to drop out. A total of 565 participants completed all three of the Web surveys (preprogram, postprogram, and 6-month follow-up) for a response rate of 48%. Participants were predominantly female (86%) and middle-aged (59% were aged 35 to 54 years). A total of 82% identified themselves as white, 7% as Asian, and 4% as African American. Most were nonsmokers (92%).

At baseline, 24% of participants were sedentary (23% were planning to start becoming active), and 36% participated in at least some physical activity but less than that recommended by the CDC guidelines (Table 2). Increases in physical activity at the 10-week follow-up were large and statistically significant. Those who were sedentary decreased from 23% to 6% (*P* < .001), and those meeting the CDC guidelines increased from 34% to 48% (*P* < .001). The percentage exercising long enough to work up a sweat often or sometimes increased from 76% to 91% (*P* < .001). Exercise metabolic equivalents increased by 27% from 35.2 to 44.7 METS units (*P* = .04).

When the 6-month follow-up survey results were compared with baseline results, the proportion that were sedentary was decreased to 19%; the proportion meeting the CDC guidelines had increased to 39%, and exercise METS had decreased to 33.1 METS units (Table 2). None of these 6-month measures was significantly different from baseline except the proportion exercising enough to work up a sweat, which increased from 76% at baseline to 83% at 6 months (*P* = .005).

Almost half of the respondents (46%) reported eating at least 5 servings of fruits and vegetables per day at baseline. This proportion increased to 73% at the 10-week follow-up and remained increased at the 6-month follow-up (*P* < .001). Body mass index (BMI) remained unchanged. At baseline, 81% of employees agreed or strongly agreed that they were satisfied with Group Health as a place to work. This high level of satisfaction persisted unchanged at 10 weeks and at 6 months. The proportion of employees who agreed or strongly agreed that Group Health encouraged its employees to have a healthy lifestyle increased from 47% at baseline to 63% at 10 weeks (*P* < .001); this finding persisted at 6 months (62%; *P* < .001).

The most important benefits of participation were 1) feeling better overall (ranked in the top three by 67% and number one by 40%) and 2) having increased energy (ranked in the top three for 53% and number one for 18%). Setting a personal goal, signing up and making a commitment, and having a pedometer to track steps were the three most highly ranked motivating factors. Having a busy work or home schedule and going on vacation were the most important barriers.

In all, 370 employees provided comments (47% of the 781 total 10-week follow-up responses). Most comments were supportive of the program (e.g., "I really enjoyed this program." "It kept me motivated, and it really helped that most of my coworkers were involved."). Several participants shared success stories of losing considerable weight or dramatically changing their physical activity and nutrition levels (e.g., "It was nice to participate, and it encouraged me to exercise. At the end, I lost weight and I was able to go on a 2-mile uphill hike. Walking up the hill had been very hard on me before."). In one of the administrative centers, some of the meetings between small groups were changed to walking meetings. Not all comments were positive. Some participants had problems with the pedometers (n = 53), and several requested better exercise facilities and benefits (n = 26).

## Interpretation

ACS's AFL was implemented at 10 Group Health facilities. At the end of the program, participants reported substantial increases in physical activity, and three quarters of those who had been sedentary at baseline were engaging in at least some moderate activity; however, at the 6-month follow-up, physical activity declined toward baseline levels.

Enrollment at Group Health (32%) was lower than that reported by Hammond (5) for the Director's Challenge (64%). Enrollment was higher at smaller clinics (52%) and the smaller administrative center (93%) than at the larger hospital (18%) and larger administrative center (32%). In the smaller facilities, the site captain and teams were all part of the same community, whereas in the large facilities the site captains, team captains, and teams did not necessarily know or work with the other participants. Hammond describes active involvement of CDC leaders in the Director's Challenge, and although several of Group Health's leaders participated, it was not a key element of the Group Health AFL program. Enrollment might have been increased by having more site captains, one in each work department or area in larger facilities, and more pivotal use of organizational and departmental leaders.

AFL participants were primarily white (82%) and female (86%). Women occupy most health care support positions, and the percentage of women in the AFL program mirrors national statistics (11) and those for the Seattle Metropolitan Area, where 86% of residents self-report as white (12), so AFL participants were broadly representative of both health care workers and the state. Most participants (81%) were satisfied with Group Health as a place to work, and it is possible that less satisfied employees would have responded differently to the program. The belief that the organization cared about its employees' health increased and this finding persisted at the 6-month follow-up.

More than half of the participants (51%) reported points for all 10 weeks of the program. Barriers to AFL goal attainment included busy work and home schedules and vacations (AFL was implemented during the summer). Participants were more likely to drop out if team captains had difficulty collecting points. Team captains complained about the time required to collect and tally team participant points (approximately 2 hours per week). If team captains had been relieved of this responsibility, they may have had more time to focus on social support (problem solving, sharing success stories, and team activities). ACS has since changed the format of its program so that participants enter points electronically.

The evaluation of this pilot had some important limitations. Lack of a control group weakened internal validity. Participation was voluntary, and those who chose to participate may have been healthier and more motivated to increase their physical activity. Additionally, all outcome variables were self-reported, and there were no objective measures of activity or fitness. Respondents to the survey may have been more successful with the program than those who dropped out. Dropouts may not have understood that they needed to complete follow-up surveys; however, 82% of those who completed the 10-week survey also completed the 6-month follow-up, making it less likely that the 6-month findings were influenced by survey-response bias.

Ongoing support for programs such as AFL may be required for lasting behavior change. In a recent study, Proper (13) found that ongoing one-on-one motivational counseling at the worksite increased physical activity at the 9-month follow-up. In a study by Heirich et al (14), providing social support in the form of support groups or motivational counselors, alone or in combination with access to fitness facilities or organized activities, resulted in increased levels of physical activity at the 3-year follow-up, whereas providing either educational classes or fitness facilities without social support did not. This finding suggests that education or environmental change alone is not sufficient. Others have found that environmental changes, such as point-of-decision prompts, increase stair use, but whether this measure increases physical activity levels overall or sustains them over time remains unknown (4,15). Continued use of larger economic incentives might lead to more lasting changes, but we found no studies that have used these alone to increase physical activity at the worksite (15). Future interventions might include extending the length of the program, repeating the program, or providing larger economic incentives over longer periods of time. Any such alternative models should be carefully evaluated, using a randomized design if possible.

## Figures and Tables

**Table 1:** Timeline of Activities for Group Health's Active for Life (AFL) Pilot

Date	Activity	People Involved
2003	Alliance for Reducing Cancer Northwest meeting	Group Health Associate Director of Prevention, American Cancer Society (ACS) representative, committee members
2/2004	Sponsorship obtained to begin planning the project	Group Health executive officer group
3/2004	Program planning begins; start-up meeting to establish timeline and committee roles; Web development of enrollment forms, point sheets, healthy lifestyle information; evaluation plan developed	Project committee consisting of members from the following Group Health departments: Clinical Information and Education; Informatics; Marketing and Communications; Prevention; and ACS representatives
3/20/2004	Invitation sent to 10 selected sites	Administrators at selected sites
4/2004-5/2004	Site captains selected and trained by an ACS representative (2 hours of telephone meetings)	One site captain for each of the clinics, hospitals, and administrative sites
5/2004	E-mail message from the medical director and chief executive officer about AFL, encouragement to participate, and links to internal Web site for enrollment	Marketing and Communications, leadership
4/04/2004- 5/21/2004	Continued advertising and promotion by intranet e-mail, newsletters, local activities, and word of mouth	Project committee, communications, Web developer, site captains
5/21/2004-5/27/2004	Online registration closes; participants receive pedometers; site captains complete formation of teams and assign team captains	Project committee, site captains, team captains, participants
6/7/2004	AFL begins 10-week program	
5/27/2004- 6/10/2004	Baseline Assessment done via internal e-mail and Survey Monkey	Project committee, prevention, ACS, participants
6/04/2004- 8/04/2004	Team captains collect weekly points; site captains send team scores to project team	Project committee, site captains, team captains, participants
6/2004- 8/2004	Weekly e-mail with updates	Project committee and site captains
8/15/2004	AFL ends; small incentives sent to all participants: team captains receive T-shirts, site captains receive T-shirts and cookbooks, team and individual prizes awarded	Project committee, site captains, team captains, participants
8/15/2004- 9/2/2004	10-week assessment	Project committee, prevention, ACS
11/29/2004-2/17/2004	6-month assessment	Project committee, prevention, ACS

**Table 2:** Physical Activity at Baseline and 10-Week and 6-Month Follow-up Among Participants in Group Health's Active for Life Program

**Current Level of Physical Activity**	**Baseline (n=565)**	**10 Weeks** **(n=565)**	**Chi-Square Comparison Between Baseline and 10 Weeks, *P* Value**	**6 Months (n=565)**	**Chi-Square Comparison Between Baseline and 6 Months, *P* Value**
**Stage-of-change question^a^**					
Sedentary	23	6	<.001	19	.15
No plans to exercise	0	1	<.001	1	.09
Thinking about starting to exercise	23	5		19	
Moderate exercise, <4 days per week	36	30		35	
Moderate exercise, ≥4 days per week for <6 months	10	27		8	
Moderate exercise, ≥4 days per week for ≥6 months	30	37		37	
**Godin Weekly Leisure-Time Exercise Questionnaire^b^**					
METs^c^ per week	35.2	44.7	.04	33.1	.54
Exercised moderately or strenuously 3-5 times per week^d^	34	48	<.001	39	.10
During typical week, engaged in regular activity long enough to work up a sweat	76	91	<.001	83	.005

Numbers indicate percentage of participants unless otherwise indicated. MET indicates metabolic equivalent.

a The stage-of-change question included the following possible responses: 1) I don’t exercise or walk regularly now, and I have no plans to start; 2) I don’t exercise or walk regularly now, but I’ve been thinking about starting; 3) I have been exercising or walking regularly (at a moderate pace or more) fewer than 4 days a week; 4) I have been exercising or walking regularly (at a moderate pace or more) at least 4 days a week for less than 6 months; 5) I have been exercising or walking regularly (at a moderate pace or more) at least 4 days a week for 6 months or longer.

b The Godin Weekly Leisure-Time Exercise Questionnaire includes the following question: “Considering a 7-day period (a week), how many times on average do you do the following kinds of exercise for more than 15 minutes during your free time?” Possible responses include the following: 1) strenuous activity (heart beats rapidly, difficult to hold a conversation [e.g., running, aerobics); 2) moderate activity (not exhausting, can have a conversation [e.g., fast walking, easy swimming, bicycling at a moderate pace]); 3) mild exercise (minimal effort [e.g., easy walking, golf, bowling, fishing, yoga, light gardening).

c Weekly METs = (frequency of strenuous exercise × 7) + (frequency of moderate exercise × 5) + (frequency of mild exercise × 3).

d Participant exercised moderately for at least 15 minutes 5 times per week or more or strenuously for at least 15 minutes 3 times per week or more.
